# Comparative impact of persistent and paroxysmal atrial fibrillation on myocardial strain: a retrospective cohort study

**DOI:** 10.1186/s40001-025-03224-9

**Published:** 2025-10-10

**Authors:** Hongru Chen, Lei Cheng, Wen Gao, Guoqian Huang, Yikai Zhao, Jian Li

**Affiliations:** Department of Cardiology, Huashan Hospital, Fudan University, No. 12 Middle Wulumuqi Road, Jing’an District, Shanghai, China

**Keywords:** Atrial fibrillation, Myocardial strain, Arrhythmia

## Abstract

**Background:**

Atrial fibrillation (AF) impairs cardiac structure and function, increasing adverse cardiovascular outcomes. Myocardial strain analysis provides a sensitive measure of myocardial deformation and has emerged as a valuable tool in detecting subclinical cardiac dysfunction. However, comparative data on myocardial strain between paroxysmal AF (PAF) and persistent AF (PsAF) remain limited. This study aimed to investigate the independent effects of different AF subtypes on myocardial strain.

**Methods:**

We conducted a retrospective analysis of 128 non-valvular AF patients between January 2023 and January 2025. Patients were classified into PAF (n = 77) and PsAF (n = 51) groups based on preoperative 24-h Holter monitoring. Speckle-tracking echocardiography was used to assess myocardial strain. Multivariate linear regression models were constructed to evaluate the association between AF subtype and the global longitudinal peak strain average (GLPS_AVG). Subgroup analyses were conducted across age, gender, BMI, hypertension, hypertriglyceridemia and diabetes.

**Results:**

Patients with PsAF exhibited significantly lower GLPS_AVG values compared to those with PAF (− 14.00 ± 3.24 vs. − 17.71 ± 3.07, P < 0.001). In multivariable-adjusted models, PsAF remained independently associated with reduced GLPS_AVG (Model III: β = 2.70, 95% CI 1.11 ~ 4.29, P < 0.001). Subgroup analysis confirmed that this association exists in every subgroups.

**Conclusion:**

Persistent AF is associated with more severe impairment in myocardial strain compared to paroxysmal AF. These findings suggest that myocardial strain analysis may aid in early detection of AF-related cardiac dysfunction and support the need for timely intervention in patients with PsAF.

## Introduction

Atrial fibrillation (AF), one of the most common sustained cardiac arrhythmias encountered in clinical practice, has demonstrated a marked rise in global prevalence [1]. It is significantly associated with increased risks of both stroke and heart failure, thereby profoundly impairing patients’ quality of life [2, 3].

Recent studies have revealed that AF-induced cardiac remodeling evolves dynamically: electrical remodeling begins with abbreviation of the atrial effective refractory period and downregulation of L-type calcium currents, which facilitate reentrant arrhythmias; structural remodeling is characterized by progressive atrial dilation and interstitial fibrosis, establishing a vicious “AF begets AF” cycle [4, 5]. This bidirectional remodeling not only compromises atrial function but also, through ventricular rate irregularity and neurohumoral activation, disrupts ventricular mechano-electrical coupling and ultimately precipitates atrial cardiomyopathy and tachycardia-induced cardiomyopathy [6]. However, conventional echocardiographic parameters such as left ventricular ejection fraction (LVEF) are markedly limited in early detection of myocardial dysfunction: up to 30% of AF patients maintain a normal LVEF at the time of clinical presentation [7]. Breakthroughs in myocardial strain analysis have provided a novel paradigm for precisely quantifying AF-related myocardial impairment. Three-dimensional speckle-tracking echocardiography (3D-STE) allows quantitative assessment of myocardial deformation in longitudinal, circumferential, and radial dimensions; its sensitivity for detecting subclinical myocardial dysfunction exceeds that of traditional metrics by over 40% [8]. Recent evidence has demonstrated that peak atrial longitudinal strain independently predicts AF recurrence [9], while each 1% decrement in global longitudinal strain of the left ventricle is associated with an 11% increase in risk of major adverse cardiovascular events [10]. Notably, AF subtype may influence strain trajectories via differences in arrhythmia burden—PAF exhibits intermittent electromechanical dissociation, whereas persistent PsAF imposes sustained hemodynamic stress, potentially leading to distinct patterns of ventricular remodeling.

Although existing evidence suggests that clinical myocardial strain parameters hold significant value in AF management, most studies to date have focused on comparisons between AF and sinus-rhythm cohorts, and a systematic characterization of the divergent mechanical-remodeling features in PAF versus PsAF is still lacking. Accordingly, this study will employ a retrospective cohort design combined with advanced myocardial strain-analysis techniques to systematically delineate the differences in myocardial mechanics between PAF and PsAF patients, thereby providing a theoretical foundation for the early identification and intervention of AF-related cardiac dysfunction.

## Materials and methods

### Data source and study population

This cohort study analyzed patients with atrial fibrillation who underwent their first radiofrequency ablation in the Department of Cardiology at Huashan Hospital, Fudan University, between January 2023 and January 2025 Clinical data were collected, and patients were categorized into paroxysmal and persistent atrial fibrillation groups based on preoperative 24-h Holter electrocardiogram results.

This study enrolled AF patients aged 18–85 years who were diagnosed with non-valvular had adequate preoperative anticoagulation, and met the indications for radiofrequency ablation. All participants completed 24-h Holter monitoring, routine transthoracic echocardiography, and speckle-tracking echocardiography during preoperative preparation. Exclusion criteria included participants with contraindications to radiofrequency ablation for AF, individuals experiencing a recurrence of AF after radiofrequency ablation, and those with conditions such as acute myocardial infarction, hypertrophic obstructive cardiomyopathy, decompensated heart failure, malignancies, hyperthyroidism, pregnancy, or severe hepatic or renal insufficiency. A total of 128 participants were enrolled in this study.

### Myocardial strain

Myocardial strain (MS) refers to the myocardium deformation during the cardiac cycle, defined as the percentage change in myocardial length relative to its initial or unstressed state, it characterizes the myocardial tissue’s elongation or shortening, as well as the thickening or thinning [11]. Based on the heart's complex three-dimensional anatomical structure, myocardial strain can be classified into three types according to the direction of deformation: longitudinal strain, circumferential strain, and radial strain [8]. Compared with LVEF, myocardial strain is more sensitive in quantifying global and regional myocardial contractile function, offering significant advantages in early detecting myocardial injury and metabolic abnormalities [12]. In patients with valvular heart disease, hypertension, or those receiving potentially cardiotoxic chemotherapeutic agents, myocardial strain can identify subclinical cardiac dysfunction before any decline in LVEF is observed [13–15]. Furthermore, in myocardial infarction patients, myocardial strain delineates the extent and severity of infarction, assists in establishing diagnostic and therapeutic strategies, and evaluates patient prognosis and treatment efficacy [16]. Additionally, strain analysis is an effective tool for assessing ventricular wall motion dyssynchrony and has predictive value for cardiac resynchronization therapy outcomes [17]. These characteristics render myocardial strain essential for guiding early intervention and preventing irreversible myocardial damage.

**Fig. 1:**
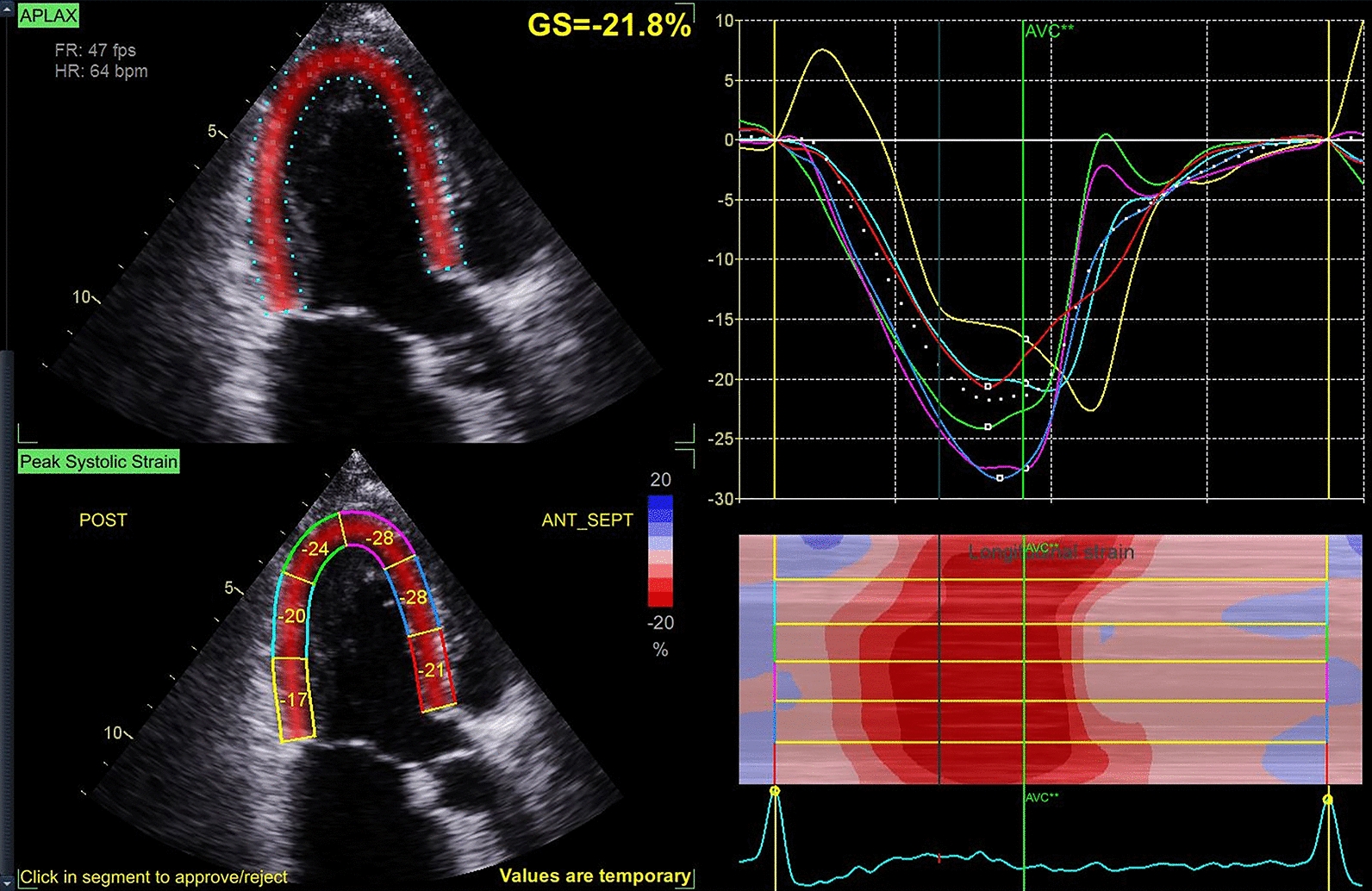
Myocardial strain analysis

In this study, our primary focus was assessing and measuring myocardial longitudinal strain. The Global Longitudinal Peak Strain Average (GLPS_AVG) was calculated as the arithmetic mean of GLS values from the apical 4-, 2-, and 3-chamber views, representing the overall level of longitudinal strain [18, 19]. For each apical view (4-, 2-, and 3-chamber), we measured GLPS on five consecutive stable beats, excluding the first beat following a long RR interval or any ectopic beat, in accordance with ASE/EACVI recommendations [20]. For paroxysmal AF patients, GLPS was measured exclusively during sinus rhythm. Any beat occurring during AF was excluded and replaced to maintain a five-beat sequence. The average GLPS of these five beats within each view was calculated, and GLPS_AVG was then obtained by taking the mean of the three view-specific averages. Relevant parameters were obtained using speckle-tracking echocardiography, with image acquisition performed by a highly experienced echocardiographer. All ultrasound image acquisition and measurements were conducted following the current guidelines of the American Society of Echocardiography. The acquired images were imported into a dedicated offline analysis workstation (EchoPAC version 2.0), where two additional investigators analyzed the image data. Each investigator performed two analyses per image to mitigate intra-observer variability, and the final evaluation was based on the average of the measurements from the two investigators to minimize inter-observer differences. If any analyzed parameter exceeded 10 standard deviations, the image was re-analyzed and re-evaluated. GLPS (global longitudinal peak strain) reflects left ventricular systolic function and is typically expressed as a negative value (shortening during contraction yields a negative strain), with larger absolute values indicating better contractile function (Fig. 1).

### Baseline data collection

Preoperative objective data, including gender, age, BMI (Body mass index) and comorbidities such as hypertension, hypertriglyceridemia, diabetes mellitus, were collected. Relevant laboratory and auxiliary examination data were also gathered, including hemoglobin (Hb), potassium (K⁺), hemoglobin A1c (HbA1c), N-terminal pro-B-type natriuretic peptide (NT-proBNP), cardiac troponin T (Ctnt), uric acid (UA), triglycerides (TG), total cholesterol (TC), low-density lipoprotein cholesterol (LDL-C), thyroid-stimulating hormone (TSH), etc. Echocardiographic and speckle-tracking echocardiographic parameters were evaluated, including left atrial anteroposterior diameter (LAD), left ventricular ejection fraction (LVEF), global longitudinal peak strain average (GLPS_AVG), Left ventricular internal dimension at end-diastole(LVIDd), Left ventricular internal dimension at end-systole(LVIDs), Left ventricular posterior wall thickness at end-diastole(LVPWd), the ratio of peak early to late diastolic filling velocity(E/A), the ratio of transmitral E velocity to early diastolic mitral annular velocity(E/e’), the heart rate was recorded during each examination, and the mean value of heart rates measured in the A4C, A2C, and A3C views was used as the representative HR value for each patient.

### Statistical analysis

Statistical analyses were performed using R software version 4.3.0. Normally distributed continuous variables are expressed as mean ± standard deviation (x̄ ± s) and were compared between groups using independent t-tests. Non-normally distributed continuous data are presented as median (Q1, Q3) and compared using the Mann–Whitney U test. Categorical variables are reported as frequencies (%) and were analyzed using the chi-square test or Fisher’s exact test. Univariate regression analyses were conducted to assess the influence of potential predictors on GLPS_AVG. Variables that reached statistical significance in the univariate analyses were subsequently incorporated into a multivariate regression model. Multivariate linear regression three models were constructed: Model I represents the unadjusted analysis; Model II adjusts for gender and age; and Model III further adjusts for additional factors, including diabetes, hypertension, hypertriglyceridemia, BMI, LVIDd, LVIDs, LVPWd, LVEF, LAD, E/A, E/e’, Hb, HbA1c, NT-proBNP, cTnT, K⁺, UA, TC, LDL-C, TG, TSH. Furthermore, subgroup analyses were performed for covariates such as age, gender, BMI, hypertension, hypertriglyceridemia and diabetes. *P* values < 0.05 were considered statistically significant.

## Results

### Baseline characteristics of study participants

A total of 128 participants were enrolled in this study, comprising 77 individuals in the paroxysmal atrial fibrillation group and 51 in the persistent atrial fibrillation group. There were no statistically significant differences between the two groups in baseline characteristics such as Age, Gender, Diabetes, Hypertension, Hypertriglyceridemia, Heart rate, BMI, LVIDd, LVIDs, LVPWd, E/A, E/e´, Hb, K⁺, HbA1c, UA, TG, LDL-C, TC and TSH (P > 0.05). However, significant differences were observed in NT-proBNP, cTnT, LAD, GLPS_AVG and LVEF (*P* < 0.05) (Table 1).

**Table 1:** Baseline characteristics between patients with paroxysmal and persistent Atrial Fibrillation

Variables	All patients (n = 128)	PAF group (n = 77)	PsAF group (n = 51)	*P*
Age, years	63.50 (55.00, 70.00)	62.00 (53.00, 68.00)	64.00 (58.00, 71.00)	0.132
Gender, n (%)				0.063
Female	34 (26.56)	25 (32.47)	9 (17.65)	
Male	94 (73.44)	52 (67.53)	42 (82.35)	
Diabetes, n (%)	25 (19.53)	15 (19.48)	10 (19.61)	0.986
Hypertension, n(%)	59 (46.09)	34 (44.16)	25 (49.02)	0.589
Hypertriglyceridemia, n (%)	52 (40.62)	32 (41.56)	20 (39.22)	0.792
Heart rate, bpm	71.50 ± 6.83	70.83 ± 6.18	72.51 ± 7.67	0.174
BMI, kg/m^2^	25.44 ± 3.17	25.58 ± 2.95	25.24 ± 3.49	0.557
Hb, g/L	141.35 ± 14.70	140.13 ± 14.25	143.20 ± 15.33	0.250
K⁺, mmol/L	4.00 ± 0.31	4.03 ± 0.31	3.96 ± 0.31	0.349
HbA1c, %	5.90 (5.60, 6.30)	5.90 (5.60, 6.30)	5.80 (5.65, 6.30)	0.864
NT-proBNP, pg/ml	139.25 (48.08, 556.73)	75.20 (34.10, 135.45)	683.80 (375.40, 1207.00)	** < 0.001**
Ctnt, ng/ml	0.007 (0.005, 0.011)	0.006 (0.005, 0.009)	0.009 (0.007, 0.013)	** < 0.001**
UA, mmol/L	0.35 (0.28, 0.42)	0.35 (0.27, 0.42)	0.35 (0.30, 0.40)	0.418
TG, mmol/L	1.42 (0.92, 2.03)	1.47 (0.95, 2.01)	1.24 (0.82, 2.05)	0.301
LDL-C, mmol/L	2.44 ± 0.84	2.47 ± 0.84	2.39 ± 0.84	0.576
TC, mmol/L	4.21 ± 1.01	4.27 ± 0.98	4.12 ± 1.07	0.415
TSH, mIU/L	2.01 (1.20, 2.89)	1.96 (1.11, 2.68)	2.03 (1.25, 3.05)	0.313
LAD, mm	39.37 ± 4.52	37.58 ± 3.70	42.06 ± 4.34	** < 0.001**
GLPS_AVG, %	− 16.23 ± 3.62	− 17.71 ± 3.07	− 14.00 ± 3.24	** < 0.001**
LVEF, %	63.00 (58.00, 66.00)	64.00 (61.00, 67.00)	60.00 (52.00, 64.00)	** < 0.001**
LVIDd, mm	49.408 ± 4.191	49.711 ± 4.329	48.939 ± 3.966	0.317
LVIDs, mm	32.849 ± 4.685	32.750 ± 4.671	33.000 ± 4.751	0.771
LVPWd, mm	8.452 ± 0.952	8.342 ± 0.857	8.620 ± 1.067	0.109
E/A	1.133 ± 1.258	1.158 ± 1.338	0.946 ± 0.255	0.637
E/e´	7.669 ± 1.992	7.758 ± 2.056	6.963 ± 1.257	0.290

*PAF* paroxysmal atrial fibrillation, *PsAF* persistent atrial fibrillation group, *BMI* body mass index, *Hb* hemoglobin, *K⁺* potassium, *HbA1c* glycated hemoglobin, *NT-proBNP* N-terminal pro-B-type natriuretic peptide, *Ctnt* cardiac troponin T, *UA* uric acid, *TG* triglycerides, *LDL-C* low-density lipoprotein cholesterol, *TC* total cholesterol, *TSH* thyroid-stimulating hormone, *LAD* left atrial anteroposterior diameter, *GLPS_AVG* global longitudinal peak strain average, *LVEF* left ventricular ejection fractions, *LVIDd* Left ventricular internal dimension at end-diastole, *LVIDs* Left ventricular internal dimension at end-systole, *LVPWd* Left ventricular posterior wall thickness at end-diastole, *E/A* the ratio of peak early to late diastolic filling velocity, *E/e*’ the ratio of transmitral E velocity to early diastolic mitral annular velocity

Association between atrial fibrillation types and GLPS_AVG.

Univariate regression analysis showed that PsAF, Male, LAD, NT-proBNP, UA, LVEF and TC were significantly associated with GLPS_AVG (*P* < 0.05) (Table 2). After including the above variables in multivariable regression analysis, it was found that PsAF and TC were significantly associated with GLPS_AVG (*P* < 0.05) (Table 3).

**Table 2:** Univariate regression analysis

Variables	β	SE	t	*P*	β (95%CI)
Types					
PAF					0.00 (Reference)
PsAF	3.71	0.57	6.54	** < 0.001**	3.71 (2.60 ~ 4.82)
Gender					
Female					0.00 (Reference)
Male	2.56	0.69	3.71	** < 0.001**	2.56 (1.21 ~ 3.92)
Age	− 0.03	0.03	− 1.13	0.260	− 0.03 (− 0.09 ~ 0.02)
BMI	0.19	0.10	1.90	0.060	0.19 (− 0.01 ~ 0.39)
LVIDd	− 0.11	0.08	− 1.38	0.170	− 0.11 (− 0.26 ~ 0.04)
LVIDs	− 0.00	0.07	− 0.05	0.957	− 0.00 (− 0.14 ~ 0.13)
IVSd	− 0.10	0.09	− 1.10	0.272	− 0.10 (− 0.28 ~ 0.08)
LVPWd	0.32	0.34	0.96	0.340	0.32 (− 0.34 ~ 0.99)
E/A	− 0.27	0.23	− 1.19	0.238	− 0.27 (− 0.71 ~ 0.17)
E/e´	0.11	0.15	0.72	0.476	0.11 (− 0.18 ~ 0.40)
LVEF	− 0.09	0.03	− 2.80	**0.006**	− 0.09 (− 0.16 ~ − 0.03)
LAD	0.31	0.07	4.79	** < 0.001**	0.31 (0.19 ~ 0.44)
Hb	0.04	0.02	1.89	0.061	0.04 (− 0.00 ~ 0.08)
HBA1C	0.31	0.46	0.66	0.508	0.31 (− 0.60 ~ 1.21)
NT-proBNP	0.01	0.00	3.61	** < 0.001**	0.01 (0.01 ~ 0.01)
Ctnt	0.95	2.58	0.37	0.714	0.95 (− 4.11 ~ 6.00)
K⁺	− 0.66	1.02	− 0.65	0.519	− 0.66 (− 2.65 ~ 1.34)
UA	7.63	3.03	2.52	**0.013**	7.63 (1.68 ~ 13.57)
TC	− 0.71	0.31	− 2.26	**0.026**	− 0.71 (− 1.32 ~ − 0.09)
LDL-C	− 0.48	0.39	− 1.26	0.211	− 0.48 (− 1.24 ~ 0.27)
TG	− 0.02	0.21	− 0.11	0.911	− 0.02 (− 0.43 ~ 0.38)
TSH	− 0.03	0.06	− 0.42	0.676	− 0.03 (− 0.15 ~ 0.10)

*CI* Confidence interval

**Table 3:** Multivariate regression analysis

Variables	β	S.E	t	*P*	β (95%CI)
Types					
PAF					0.00 (Reference)
PsAF	2.85	0.73	3.89	** < 0.001**	2.85 (1.41 ~ 4.28)
Gender					
Female					0.00 (Reference)
Male	1.07	0.67	1.60	0.113	1.07 (− 0.24 ~ 2.38)
LVEF	− 0.03	0.03	− 1.05	0.297	− 0.03 (− 0.09 ~ 0.03)
LAD	0.12	0.07	1.79	0.077	0.12 (− 0.01 ~ 0.26)
NT-proBNP	− 0.00	0.00	− 0.43	0.669	− 0.00 (− 0.00 ~ 0.00)
UA	5.38	2.81	1.92	0.058	5.38 (− 0.12 ~ 10.88)
TC	− 0.58	0.28	− 2.06	**0.042**	− 0.58 (− 1.14 ~ − 0.03)

*CI* Confidence interval

Multivariate regression models were constructed to evaluate the association between AF subtype and GLPS_AVG. In the unadjusted model (Model I), persistent AF was associated with significantly worse GLPS_AVG compared to paroxysmal AF (β = 3.71, 95% CI 2.60–4.82, P < 0.001). This difference persisted after adjustment for sex and age in Model II (β = 3.61, 95% CI 2.49–4.73, P < 0.001). In Model III, which further included diabetes, hypertension, hypertriglyceridemia, BMI, LVIDd, LVIDs, LVPWd, LVEF, LAD, E/A, E/e’, Hb, HbA1c, NT-proBNP, cTnT, K⁺, UA, TC, LDL-C, TG, and TSH, persistent AF remained independently associated with worse GLPS_AVG (β = 2.70, 95% CI 1.11–4.29, P < 0.001) (Table 4).

**Table 4:** Comparison of regression analysis results for PAF and PsAF across different models

Variables	Model I		Model II		Model III	
	β (95%CI)	*P*	β (95%CI)	*P*	β (95%CI)	*P*
Types						
PAF	0.00 (Reference)		0.00 (Reference)		0.00 (Reference)	
PsAF	3.71 (2.60 ~ 4.82)	** < 0.001**	3.61 (2.49 ~ 4.73)	** < 0.001**	2.70 (1.11 ~ 4.29)	** < 0.001**

*CI* Confidence interval

Model I: None

Model II: Adjust: Gender, Age

Model III: Adjust: Gender, Age, Diabetes, Hypertension, Hypertriglyceridemia, BMI, LVIDd, LVIDs, LVPWd, LVEF, LAD, E/A, E/e’, Hb, HbA1c, NT-proBNP, cTnT, K⁺, UA, TC, LDL-C, TG, TSH

### Subgroup analysis

**Fig. 2:**
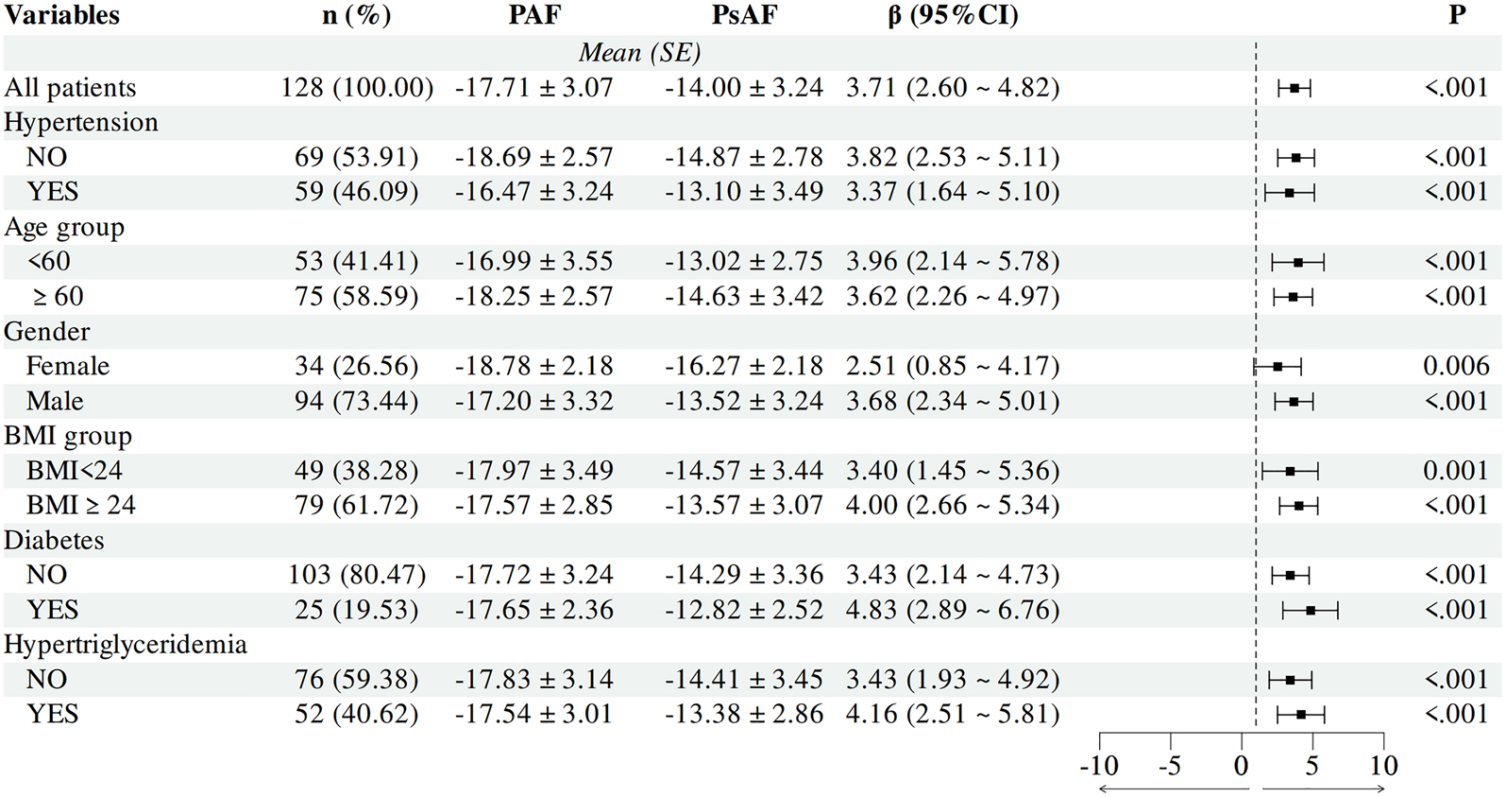
Subgroup analysis

This study compared the differences in GLPS_AVG between patients with persistent atrial fibrillation (PsAF) and paroxysmal atrial fibrillation (PAF) across various subgroups, including age, gender, BMI, hypertension, hypertriglyceridemia and diabetes. In every subgroup, PsAF patients exhibited significantly worse GLPS_AVG than PAF patients (P < 0.05) (Fig. 2).

## Discussion

This study is to compare the effects of paroxysmal and persistent atrial fibrillation on myocardial longitudinal strain, In this observational cohort study, we found that myocardial longitudinal strain is more severely impaired in PsAF patients than in PAF patients. Specifically, based on the comparison of baseline data, the longitudinal strain parameters of PsAF patients were significantly worse than those of PAF patients. Even after sequential adjustment for demographic factors, cardiac structural parameters, biomarkers, and other potential confounders, the global longitudinal peak strain average (GLPS_AVG) in the PsAF group remained significantly worse than in the PAF group. Moreover, subgroup analysis confirmed that this difference persisted across patient different subpopulations, suggesting that PsAF may induce more pronounced cardiac dysfunction than PAF.

The observed greater impairment of GLPS_AVG in PsAF patients may be related to progressive cardiac remodeling induced by a persistent irregular rhythm. Persistent atrial fibrillation leads to electrical and structural remodeling of the heart, including atrial dilation, myocardial fibrosis, and ventricular dyssynchrony, impairing myocardial systolic and diastolic function together [21, 22]. The reduction in GLPS_AVG in PsAF patients may reflect these maladaptive changes, as myocardial strain is susceptible to early subclinical dysfunction. Notably, the difference remained significant after multivariable adjustment (Model III: β = 2.67, P < 0.001), further supporting that persistent AF, rather than confounding comorbidities, is the primary driver of worsened myocardial mechanics. Furthermore, the results of subgroup analyses support the generalizability of these associations, reinforcing that the negative impact of persistent AF on myocardial mechanics is more pronounced than that of paroxysmal AF across different populations.

The multivariable model presented in Table 3 indicated that lower serum total cholesterol (TC) levels were associated with worse GLPS_AVG values, suggesting an inverse relationship between TC and myocardial strain. Although this association appears counterintuitive, similar findings have been reported in previous cardiovascular studies. The so-called “cholesterol paradox” has been described, wherein lower lipid levels may indicate more advanced disease status in certain cardiovascular populations. Czerniak reported that low TC levels often serve as a marker of disease severity [23]. This inverse association may reflect underlying factors such as malnutrition, elevated systemic inflammation, or intensive lipid-lowering therapy. In the present cohort, lower TC levels may be indicative of older age or coexisting metabolic disorders, both of which are associated with impaired myocardial function. It is therefore possible that reduced cholesterol levels reflect a more severe systemic condition rather than directly contributing to left ventricular dysfunction.

A previous study demonstrated that persistent AF patients exhibit greater reductions in global longitudinal and circumferential strain than those with paroxysmal AF, Our results build upon and extend previous work by uncovering a pronounced impairment in left ventricular systolic synchrony using 3D-STE [24, 25]. Importantly, none of the patients in our study cohort underwent catheter ablation, thereby minimizing confounding from prior interventions. After adjustment for a comprehensive set of clinical, structural, and biochemical covariates, AF subtype remained an independent predictor of strain, providing a clear quantitative estimate of its impact. In future studies, we also intend to investigate how radiofrequency ablation influences myocardial strain in patients with atrial fibrillation.

The strengths of this study include strict adherence to the American Society of Echocardiography guidelines for image acquisition and analysis, dual-investigator evaluation to minimize observer bias, and comprehensive adjustment for confounding factors in multivariable models. However, several limitations should be acknowledged. First, the single-center design and relatively small sample size may limit the generalizability of the findings, despite averaging multiple beats per view to mitigate beat-to-beat variability in AF, residual variability may remain. Future studies might explore automated beat-selection algorithms or larger beat samples to further enhance reproducibility. Second, as an observational study, it cannot establish causal relationships. Third, we did not include long-term clinical outcomes such as heart failure, hospitalization, or mortality; therefore, prospective studies are needed to link myocardial strain parameters with clinical outcomes.

According to ASE/EACVI guidelines, this study measured GLPS based on five consecutive stable cardiac cycles, excluding beats following prolonged R-R intervals or ectopic beats. However, the strict 'index-beat' approach for cross-view beat alignment was not adopted. While this practice is consistent with many current clinical studies, the index-beat method may offer better inter-beat consistency, particularly in patients with substantial heart rate variability or arrhythmic tendencies. Given that heart rate in patients with atrial fibrillation naturally fluctuates in real-world clinical settings, such variability may still influence strain measurements despite careful selection of stable cardiac cycles. This potential measurement variability should be acknowledged, and future studies may consider incorporating the index-beat approach to further improve measurement consistency.

Prospective investigations should validate these findings in larger, multicenter cohorts and explore whether longitudinal monitoring of myocardial strain can predict clinical events or guide therapeutic strategies. Additionally, investigating the reversibility of strain parameters following a reduction in atrial fibrillation burden may help elucidate the extent to which AF-induced myocardial dysfunction is reversible.

## Conclusion

In summary, this study highlights the differential impact of AF subtypes on myocardial strain, with PsAF independently associated with more severe myocardial injury. These findings suggest that patients with PsAF may benefit from earlier and more aggressive interventions to mitigate cardiac remodeling.

## Data Availability

No datasets were generated or analysed during the current study.

## Abbreviations

AF: Atrial fibrillation
BMI: Body mass index
Ctnt: Cardiac troponin T
GLPS_AVG: Global longitudinal peak strain average
Hb: Hemoglobin
HbA1c: Hemoglobin A1c
K⁺: Potassium
LAD: Left atrial anteroposterior diameter
LDL-C: Low-density lipoprotein cholesterol
LVEF: Left ventricular ejection fractions
NT-proBNP: N-terminal pro-B-type natriuretic peptide
PAF: Paroxysmal atrial fibrillation
PsAF: Persistent atrial fibrillation group
TC: Total cholesterol
TG: Triglycerides
TSH: Thyroid-stimulating hormone
UA: Uric acid
3D-STE: Three-dimensional speckle-tracking echocardiography
